# Low-Temperature Preparation of Tungsten Oxide Anode Buffer Layer via Ultrasonic Spray Pyrolysis Method for Large-Area Organic Solar Cells

**DOI:** 10.3390/ma10070820

**Published:** 2017-07-18

**Authors:** Ran Ji, Ding Zheng, Chang Zhou, Jiang Cheng, Junsheng Yu, Lu Li

**Affiliations:** 1 State Key Laboratory of Electronic Thin Films and Integrated Devices, School of Optoelectronic Information, University of Electronic Science and Technology of China, Chengdu 610054, China; jiran1231@gmail.com (R.J.); zd901023@gmail.com (D.Z.); 2013000101001@std.uestc.edu.cn (C.Z.); 2 Co-Innovation Center for Micro/Nano Optoelectronic Materials and Devices, Research Institute for New Materials and Technology, Chongqing University of Arts and Sciences, Chongqing 402160, China; caiwu0301@163.com

**Keywords:** tungsten oxide, anode buffer layer, organic solar cells, large area, ultrasonic spray pyrolysis method

## Abstract

Tungsten oxide (WO_3_) is prepared by a low-temperature ultrasonic spray pyrolysis method in air atmosphere, and it is used as an anode buffer layer (ABL) for organic solar cells (OSCs). The properties of the WO_3_ transition metal oxide material as well as the mechanism of ultrasonic spray pyrolysis processes are investigated. The results show that the ultrasonic spray pyrolysized WO_3_ ABL exhibits low roughness, matched energy level, and high conductivity, which results in high charge transport efficiency and suppressive recombination in OSCs. As a result, compared to the OSCs based on vacuum thermal evaporated WO_3_, a higher power conversion efficiency of 3.63% is reached with low-temperature ultrasonic spray pyrolysized WO_3_ ABL. Furthermore, the mostly spray-coated OSCs with large area was fabricated, which has a power conversion efficiency of ~1%. This work significantly enhances our understanding of the preparation and application of low temperature-processed WO_3_, and highlights the potential of large area, all spray coated OSCs for sustainable commercial fabrication.

## 1. Introduction

The steady enhancement in efficiency of organic solar cells (OSCs) has made this class of photovoltaic device a highly promising technology for photo-electric conversion [1,2,3]. Recently, the state-of-the-art single junction OSCs with power conversion efficiency (PCE) approaching 12.4% hasbeen achieved [4]. To obtain OSCs with high efficiency, several approaches have been developed such as material development [5,6,7], morphology control [8,9,10,11], and device engineering [4,12,13,14]. It is well known that the performance of OSCs can be significantly improved by inserting a suitable interfacial buffer layer between active layer and electrode. The interfacial buffer layer is efficient for enhancing charge transfer efficiency, preventing photon-generated carriers from undesired recombination and modifying the electrode [15].

In the field of OSCs, an interfacial buffer layer includes cathode buffer layer and anode buffer layer (ABL), and PEDOT:PSS is mainly adopted as the ABL. Nevertheless, PEDOT:PSS is both hygroscopic and acidic, which has an associated reduction in device stability [16]. To circumvent this problem, transition metal oxides such as molybdenum oxide (MoO_3_) [16,17,18], vanadium oxide (V_2_O_5_) [19,20,21], nickel oxide (NiO) [22,23] and tungsten trioxide (WO_3_) [24,25,26,27,28] have been chosen as promising candidates as ABLs to replace PEDOT:PSS, due to their superb charge transfer capability and stability. However, most of transition metal oxides buffer layers applied in OSCs are fabricated by using vacuum thermal evaporated technique [16,17,18,28] or sol-gel methods with high temperature annealing [29,30,31], leading to increased energy consumption and low production speeds. Besides, it is much more difficult to deposit inorganic buffer materials by solution method especially on active layer, since in the high substrate temperature needed for annealing, even some of the raw materials could not be solubilized.

These are limitations for transition metal oxides for further industrial application of OSCs. Therefore, it is important to realize a path towards the commercialization of solution-processed OSCs. The development of fabrication methods should be compatible with high-volume roll-to-roll (R2R) processing techniques [32]. Among them, the ultrasonic spray pyrolysis (USP) method, which is readily scalable and open-air to produce high-quality polycrystalline metal oxide thin films, fully satisfies these demands. In addition, the USP method does not require either extended thermal anneals or additional synthetic steps [33], and several metal oxide thin films as the highly efficient transport layer have been deposited, such as zinc oxide (ZnO) [34], molybdenum trioxide (MoO_3_) [35] and tungsten trioxide (WO_3_) [36,37]. However, these WO_3_ prepared methods require high annealing temperatures (over 250 °C). 

In this work, we have used a low-decomposition-temperature ammoniumtungstate (AT) ((NH_4_)_10_W_12_O_41_) solution as a precursor to obtain a transition metal oxide material of WO_3_ thin films, which was deposited with water-based solution on top of the active layer, directly by USP method, and a mostly spray-coated OSC (without electrode) was fabricated. To illuminate the improvement of charge transport ability of spray-coated WO_3_ (S-WO_3_) ABL, the properties of composition, morphology, conductivity, and crystallinity of S-WO_3_ are investigated. Equivalent circuit model, impedance analysis and mechanism of USP coated films are also discussed. Moreover, the OSCs based on different precursor solution concentrations are optimized. To illustrate how the USP method is useful, and how S-WO_3_ is a promising candidate as ABL, we choose the OSC based on evaporated WO_3_ (E-WO_3_) as a control group, because the evaporation method is a conventional technique to fabricate WO_3_ films. The performance of S-WO_3_ ABLs based devices shows an improvement of power conversion efficiency from 3.2% to 3.6%. The result suggests that this solution processed WO_3_ is a promising anode interfacial layer for the fabrication of high efficiency OSCs. Finally, the fabrication of a large area OSC device based on S-WO_3_ ABL is performed to test the potential of large area and all spray-coated OSCs.

## 2. Results and Discussion

### 2.1.Characterization and Analysis of WO_3_ Films

**Figure 1:**
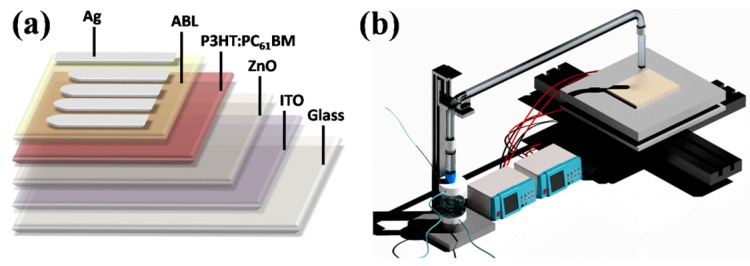
(**a**) Device architecture of inverted organic solar cell (OSC) and (**b**) Ultrasonic spray pyrolysis system.

Figure 1a shows the configuration of the photovoltaic device in this work. Figure 1b presents the device fabrication apparatus, which comprises an ultrasonic transducer, an atomization chamber, an automatic *X*-*Y* table, a heating block, and pipe fittings. Mist droplets are generated in an atomization chamber through an ultrasonic atomization of precursor solution. Then, droplets transfer through the pipe fittings and deposit on the heating block. The sprinkler is passed over the hot plate driven by the drive *X*-*Y* table and performed a snake-like curve relative motive with the substrate. 

**Figure 2:**
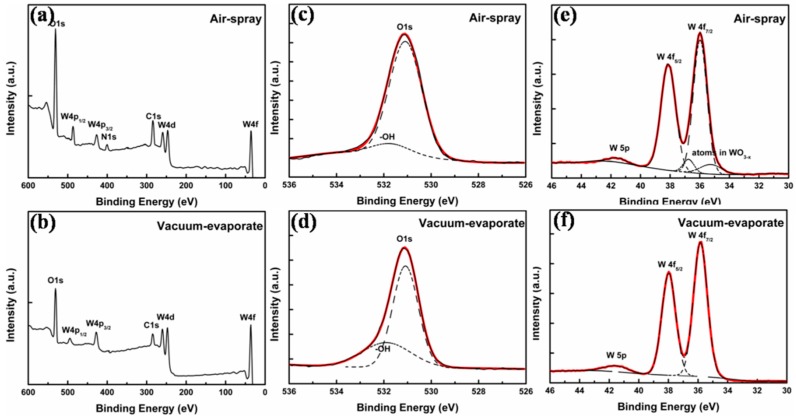
X-ray photoelectron spectroscopy (XPS) spectra of different process tungsten oxide (WO_3_) films. (**a**) Full scan; (**c**) W (4f) core levels and (**e**) O (1s) core levels of spray-coated WO_3_ (S-WO_3_) film, respectively; (**b**) Full scan; (**d**) W (4f) core levels and (**f**) O (1s) core levels of evaporated WO_3_) E-WO_3_ film, respectively.

**Figure 3:**
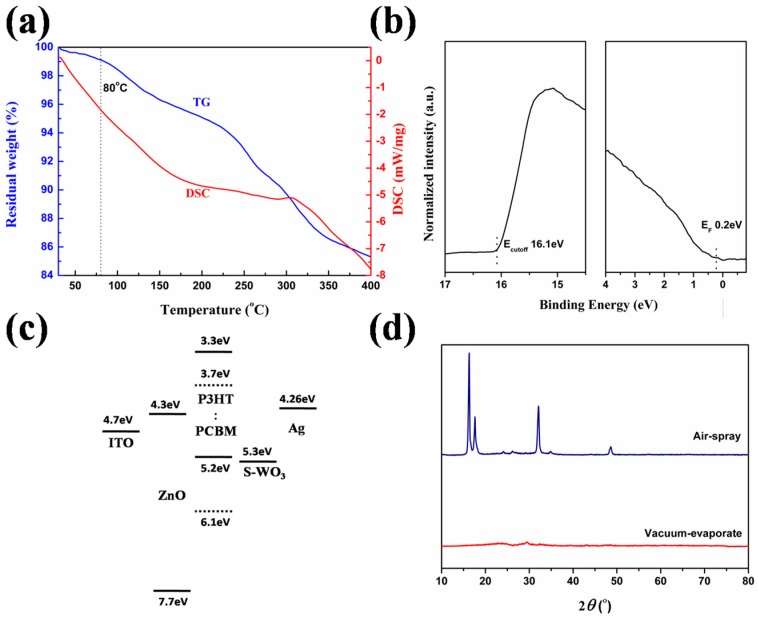
(**a**) Thermo gravimetric (TG) and differential scanning calorimeter (DSC) profiles of the AT precursor; (**b**) ultraviolet photo-electron spectroscopy (UPS) results of S-WO_3_ film; (**c**) energy level of the component materials used in the OSCs; (**d**) XRD pattern of S-WO_3_ and E-WO_3_, respectively.

To assay the elementary composition of WO_3_ films, the surface characteristics of S-WO_3_ and E-WO_3_ films on silicon wafer are characterized by X-ray photoelectron spectroscopy (XPS). The full scan spectra are shown in Figure 2a,b. O (1s) and W (4f) of two samples are characterized and shown in Figure 2c–f, respectively. The two full scan spectra show that the chemical states of S-WO_3_ and E-WO_3_ are almost identical except for an additional N 1s peak center at 400.0 ± 0.2 eV in the S-WO_3_ spectrum. It also indicates that AT does not decomposed completely under 80 °C. The thermo gravimetric analysis (TG) and differential scanning calorimeter (DSC) data as shown in Figure 3d exhibit the same result. In Figure 3d, for the dried precursor, the weight losses at 80 °C were ~4.5%, indicating that the dried precursor is the mixture of WO_3_ and AT.

Two main XPS resolved peaks are ascribed to the typical doublet of W6^+^ with binding energy of W (4f_7/2_) centered at 36.0 ± 0.2 eV. The spin orbit splitting of the doublet is 2.12 eV and the peak ratio of W (4f_7/2_) to W (4f_5/2_) is 4:3. A third broad peak of W (5p_3/2_) locates at ~42 eV. The high-resolution XPS spectrum of E-WO_3_ reveals only W6^+^ oxidation state. W atoms with an oxidation state are ascribed to WO_3_ [38]. In the S-WO_3_ film, a second doublet at 34.6 ± 0.1 eV and 36.7 ± 0.1 eV is conventionally used to fitting with the W6^+^ peak due to the asymmetry of peak at the lower binding energies. It indicates that WO_3_ readily becomes oxygen deficient to form WO_3−*x*_, with variable oxygen composition parameter *x*. This oxygen deficiency greatly influences the bulk of the electronic transport properties by introducing donor electronic states [39,40]. Additionally, the unsaturated valence of metallic W would decrease the hole injection barrier of interface when the S-WO_3_ film is contact with P3HT:PCBM [41]. Therefore, the S-WO_3_ might have a better efficiency of hole transport than E-WO_3_. 

On the other hand, the O (1s) XPS spectra of two WO_3_ samples exhibit asymmetric line shapes, and the peaks are fitted with two components. One main peak at 531.0 ± 0.1 eV corresponds to W-O bond, and the other at 532.5 ± 0.1 eV corresponds to -OH groups due to atmospheric contamination or the crystal water [42,43,44]. Obviously, there are less oxide impurities in S-WO_3_.

Then, ultraviolet photo-electron spectroscopy (UPS) is used to probe the electronic properties of S-WO_3_ which is shown in Figure 3a. We determined the work function of S-WO_3_ in the ITO/ZnO/P3HT/S-WO_3_. For the UPS measurement, a 10 nm thick S-WO_3_ film was formed on the ITO/ZnO/P3HT substrate. The UPS measurement was performed with a He I (21.2 eV) discharge lamp (AXIS–NOVA System, Kratos, Manchester, UK). The sample was kept inside a high-vacuum chamber. The work function (*WF*) is calculated from the UPS data using the following Equation (1).

(1) $$WF=h\nu +E_{cutoff}-E_{Fermi}$$

where *hν* (21.2 eV) is the incident photon energy. *E_cutoff_* (16.1 eV) is the high binding energy cutoff, and *E_Fermi_* (0.2 eV) is the valence band. Therefore, the *WF* of S-WO_3_ is 5.3 eV. The value is much lower than the publicly recognized *WF* of WO_3_ (6.7 eV), because of the formation of an interface dipole, induced by an electron transfer from organic film [45]. We think the lower *WF* value can describe the characteristics of S-WO_3_ better, which is much more suitable for S-WO_3_ in an OSC device. Moreover, as shown in Figure 3b, the matched *WF* value of S-WO_3_ allows for the formation of Ohmic contacts with a donor [46], thus resulting in the increase of the built-in field. This is beneficial for enhancing charge extraction efficiency and reducing re-combination losses [47,48].

To further disclose the physical property of WO_3_ films, the X-ray diffraction (XRD) pattern of both 20 nm-thick S-WO_3_ and 20 nm-thick E-WO_3_ films are characterized and shown in Figure 3c. It indicates that S-WO_3_ has a good crystallinity. However, no crystalline peaks are observed in E-WO_3_ film, so it can be regarded as an amorphous film.

### 2.2. Detailed Analysis of OSC Performances

**Figure 4:**
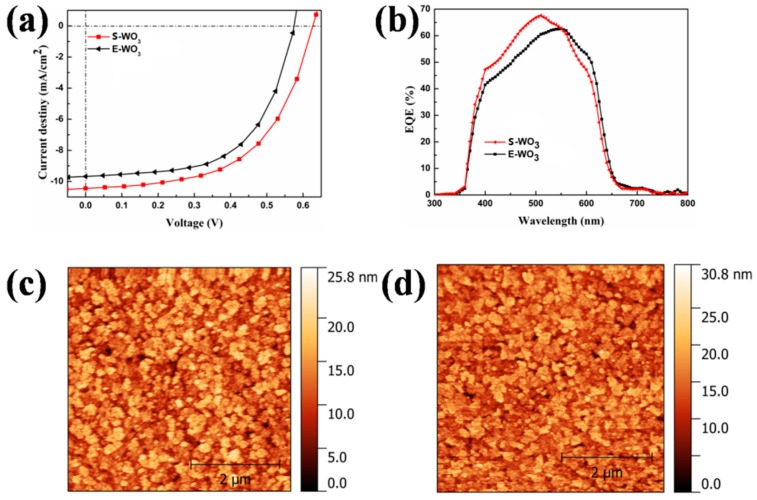
(**a**) Current density versus voltage (*J–V*) characteristics of OSCs with S-WO_3_ and E-WO_3_ films; (**b**) external quantum efficiency (*EQE*) characteristics of OSCs with S-WO_3_ and E-WO_3_ films; Atomic force microscopy (AFM) images of (**c**) S-WO_3_; (**d**) E-WO_3_.

To further investigate the influence of E-WO_3_ and S-WO_3_ with different precursor concentrations on the device performance, the OSCs were fabricated. We optimized the thickness of E-WO_3_ film, and OSCs based on 20 nm E-WO_3_which have the best performance. Therefore, we chose 20 nm as the optimal thickness of E-WO_3_. Figure 4a shows the current density versus voltage (*J–V*) characteristics of OSCs based on E-WO_3_ and S-WO_3_ films made from 100mg/L AT precursor. The *J–V* characteristics of OSCs based on S-WO_3_ made from other precursor concentrations are shown in Figure S1, and the detailed parameters are summarized in Table 1. The device using 100 mg/L AT precursor shows the highest PCE of 3.63%, which is also better than that using 20 nm E-WO_3_. The optimized device has an 11% enhancement in PCE with a simultaneous improvement in *V_OC_* (0.57 V to 0.63 V) and *J_SC_* (9.68 mA·cm^−2^ to 10.45 mA·cm^−2^). When the concentration of AT precursor solution increases from 25 to 100 mg/L, the PCE of device increases from 2.03% to 3.63% with enhancement in *V_OC_* (0.55 V to 0.63 V), *J_SC_* (9.58 mA·cm^−2^ to 10.45 mA·cm^−2^), *FF* (38.73% to 52.85%) and *R_S_* (15.65 Ω·cm^2^ to 1.18 Ω·cm^2^). Series resistances are derived from the slope of the *J–V* characteristic curve and the low *R_S_* of 1.18 Ω·cm^2^ is much comparable to the device with E-WO_3_. Once the concentration of AT precursor increases from 100 to 300 mg/L, the PCE of devices get a decrease from 3.63% to 2.86%. 

To illustrate the impact on device performance based on different ABLs, the morphology of E-WO_3_ and S-WO_3_ films made from different AT precursor concentration is characterized by atomic force microscopy (AFM). The images of E-WO_3_ and S-WO_3_ films with 100 mg/L AT precursor are shown in Figure 4. The images of substrate, active layer and S-WO_3_ films with other precursor concentrations are shown in Figure S2. Due to the surface of spray-coated active layer beingextremely rough, all films are directly fabricated on ITO to characterize the films. We find that S-WO_3_ and E-WO_3_ films have similar morphology. For the film of S-WO_3_ with the best OSC performance, a smooth surface was observed with a root mean square (RMS) of surface roughness of 3.49 nm, and even smoother than the evaporated one with a RMS of surface roughness of 3.61 nm. With the increasing of the concentration of AT precursor, the films get thicker and rougher, and the grain size of WO_3_ becomes much larger. Compared to the S-WO_3_ films, E-WO_3_ film cannot show a uniform and obvious grain. We speculate that it relates to the different crystallinity of S-WO_3_ and E-WO_3_, which confirms the result of XRD. The thicknesses of S-WO_3_ films obtained from 300 mg/L, 200 mg/L and 100 mg/L AT precursors are ~30 nm 20 nm and 15 nm, respectively. However, the S-WO_3_ films from the 25 mg/L precursor cannot cover the entire substrate. The thickness from 25 mg/L AT precursor is ~5 nm. From the perspective of device performance, it is necessary for S-WO_3_ ABL to cover the active layer fully and uniformly for reducing the leakage current. Introducing a WO_3_ layer will generally contribute to the series resistance of the device and the higher thickness of WO_3_ will block charge transport and result in a lower current [49]. Compared to the device using E-WO_3_ ABL, the devices with S-WO_3_ ABL has a much higher *V_OC_*, which is probably in consideration of the high conductivity and matching energy level of S-WO_3_ [50].

Figure 4d shows the results of external quantum efficiency (*EQE*) measurement for OSCs based on S-WO_3_ and E-WO_3_. The device spectrum based on S-WO_3_ film fabricated by 100 mg/L AT precursor shows a higher *EQE* than others, and the peak is 67.6% at a wavelength of 510 nm. The *EQE* curves of the S-WO_3_ devices with other concentration present similar shape in the entire range of wavelength between 350 and 700 nm, as shown in Figure S3. The relatively high *EQE* originates from the better Ohmiccontact acquired at the interface. We postulate that the increase of hole transport efficiency is due to the high hole mobility of S-WO_3_ ABL. The enhancement in *EQE* is also in good agreement with the improved *J_SC_*. This result indicates that the S-WO_3_ can bring an improved hole transport efficiency.

To verify our postulation, the device with a configuration of ITO/ABLs/Ag wasfabricated to demonstratethe conductivity of ABLs. The *I-V* curves of device are presented in Figure S4, and details are also provided in supplemental materials. The conductivities of E-WO_3_ and S-WO_3_ are 3.05 × 10^−4^ S∙m^−1^ and 5.17 × 10^−4^ S∙m^−1^, respectively. The conductivity of S-WO_3_ is much higher than that of E-WO_3_. These results are in good agreement with the aforementioned phenomena. This high conductivity S-WO_3_ ABL with suitable energy level can effectively block the electron and provide a well Ohmic contact between anode and active layer to enhance the hole transport efficiency of the whole device.

### 2.3. Equivalent Circuit Model and Impedance Analysis

**Figure 5:**
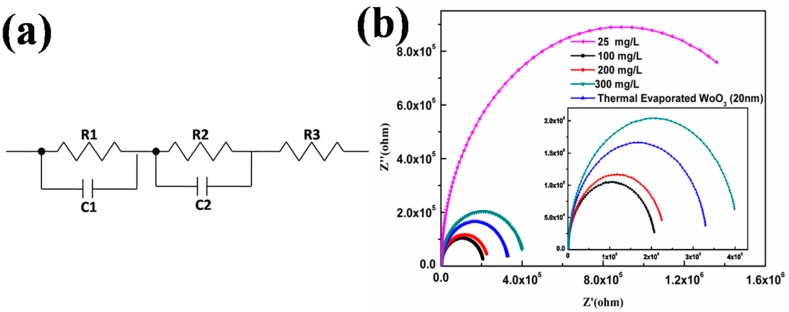
(**a**) Equivalent circuit model of the devices. (R1 and CPE1, R2 and C2, R3 represent equivalent of donor and accepter interface, interface between active layer and electrodes, resistance of electrodes, respectively; (**b**) Cole–Cole plots of the devices based on E-WO_3_ and S-WO_3_ films with different precursor concentration.

To examine the electrical contact of the interfaces in the obtained devices, a circuit model is defined according to the sandwiched device structure of OSCs as shown in Figure 5a. Figure 5b shows the nyquist plots of impedance measurement of devices based on S-WO_3_ and E-WO_3_ ABLs for frequencies ranged from 40 Hz to 5 MHz. The parallel circuit of R1 and C1 corresponds to the donor and accepter interface. R2 and C2 represent two electrical contacts of the interfaces between the active layer and electrodes. R3 represents the resistance of electrodes. Parameters employed for the fitting of the impedance spectra by using an equivalent circuit model are shown in Table 2. Compared to the device based on E-WO_3_, the R2 of the interface junction decreases from 1547 to 850 Ω when using the S-WO_3_ ABL. Moreover, the C2 of the interface junction increases from 9.60 × 10^−9^ F to 1.16 × 10^−9^ F. This indicates that the interface between the S-WO_3_ and the active layer shows a good Ohmic contact correlated to the efficient charge transport [51]. 

### 2.4. Mechanism of USP Coated Films

To realize the uniform films, especially the large area uniform ones, for large area OSCs, the mechanism of USP process should be discussed in more detail. As is well known, the atomization of precursor solution droplets should satisfy with: (1) Small droplets size; (2) Well-proportioned distribution. For USP method, the amount and diameters of droplets are related to the vapor pressure, surface tension and viscosity of precursor solution. The diameter of droplets is given by Equation (2) [52]:

(2) $$D=0.34{\left(8\pi \sigma /\rho f^{2}\right)}^{1/3}$$

where *D* is the diameter of droplet, and *σ* is the surface tension of the solvents, *ρ* is the density of solvent, and *f* is the frequency of ultrasonic wave. For a given solvent, the size of droplets is determined by the frequency. When the frequency of ultrasonic wave reached billions of hertz, the droplets with a few micron diameters would be obtained. During the spray process, droplets keep moving. The weight of droplets decreases continuously along with solvent drying, and the velocity of droplets is changed simultaneously. For a single droplet, the rate of mass change induced by evaporation is given by Equation (3) [53]:

(3) $$-\frac{dm}{dt}=2\pi RD\cdot sh\cdot (\gamma_{s}-\gamma_{\infty })$$

where *γ_s_* and *γ_∞_* are the content of solvent in ambient gas and the saturated concentration of solvent on droplet surface, respectively, and *sh* is the Sherwood constant. *D* is the diffusion coefficient of solvent, and *R* is the radius of droplet. Ultimately, the evaporation degree of droplets can be deduced by the mass change. 

**Figure 6:**
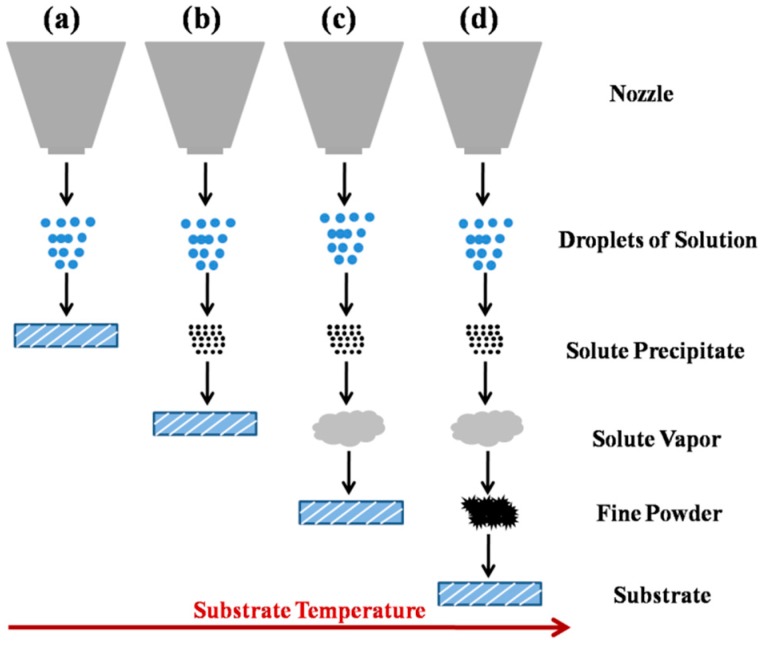
Dynamics of USP with different substrate temperature.

Viguie et al. considered that the film grown pattern of droplets pyrolytic deposition can be divided into four issues [54], which is shown in Figure 6. In the case of Figure 6a, the temperature of substrate and air atmosphere is relatively low, so solvent does not evaporated completely in air. When the droplets reach to the substrate, the solvent starts to evaporate and the solute decompose. In Figure 6b, the temperature of substrate is relatively high. As a result, before droplets reach substrate, solvent evaporates completely, and solute reaches to substrate and pyrolysis. In the condition of Figure 6c, with the increase of temperature, before droplets reach the substrate, solvent evaporates completely, and then solute melts and vaporizes. Lastly, the gaseous solute is deposited on substrate to form a film. This process is similar to chemical vapor deposition. In Figure 6d, the temperature of substrate is extremely high, the above mentioned processes of a, b, c are all completed. The end products will fall onto the substrate, leading to the forming film with poor adhesion [54]. 

In this work, the fabrication of S-WO_3_ was processed at relatively low temperature, so the processing mechanism belongs to the situation of Figure 6a: The droplets of AT precursor are deposited on the substrate, and the excess solvent is evaporated rapidly. Subsequently, partial AT decomposes into WO_3_, and the mixture grows into film. The low substrate temperature provides only a small driving force for nucleation, so the film has a low nucleation and growth rate. This means that the grain can grow much larger, resulting in smooth and uniform film [34], which is in accordance with the result of AFM image. Hence, the low temperature processing condition can yield thin uniform films with large area for potential device application. 

### 2.5. Large Area OSC

**Figure 7:**
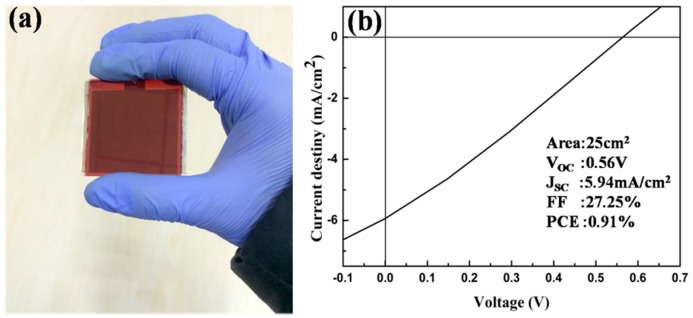
(**a**) Image of a 25 cm^2^ OSC device with as-grown S-WO_3_ film and (**b**) *J–V* characteristic of the OSC.

Based on the above discussion of USP mechanism and the analysis of OSCs performance, 100 mg/L solution of precursor is chosen as the optimal concentration to process large area uniform WO_3_ films. By using the obtained WO_3_ films, a large area OSC of 5 × 5 cm^2^ with a same structure as the above devices were successfully prepared. Figure 7a shows the image of practical OSC, and Figure 7b presents the *J–V* characteristics of the device. The *V_OC_* and the *Jsc* are 560 mV and 5.94 mA/cm^2^, respectively, indicating that this low temperature deposition method is applicable for the fabrication of large area OSCs. The PCE of the OSC with a large area of 25 cm^2^ is ~1%, which is much lower than the former OSC with a small area of 0.02 cm^2^. Among three factors to determine the PCE of OSC, the low *Jsc* of large area device is due to the large square resistance caused by the area increase of ITO electrode. The decrease of *V_OC_* and *FF* is attributed to the high leakage current with the increasing defect along with the area increase of functional films of two buffer layers and the active layer. Therefore, this low temperature USP method with the assistance of ABL can be applied for large area OSCs, but the core issue of large area devices about increasing PCE still needs to be studied. 

## 3. Materials and Methods

### 3.1. Fabrication Section

The configuration of photovoltaic device is ITO/ZnO (40 nm)/P3HT:PC_61_BM (300 nm)/ABL/Ag (100 nm). Patterned ITO-coated glass substrates with a sheet resistance of 10 Ù∙sq^−1^ were consecutively cleaned in ultrasonic bath containing detergent, acetone, deionized water and ethanol for 10 min each step, then dried by nitrogen blow [55]. Prior to the deposition of functional layers, the substrate was treated with UV light for 10 min. A 40 nm ZnO layer was spray-cast on ITO film by ultrasonic spray pyrolysis at 150 °C as depicted in our previous work [34]. Then, a 300 nm active layer of P3HT:PC_61_BM was casted with a supersonic nozzle (Z95S, Siansonic, Beijing, China) from a solution with P3HT (99.9%, Solarmer, Beijing, China) and (6,6)-phenyl-PCBM (99.9%, Solarmer, Beijing, China) at a weight ratio of 1:0.9 wt % in 1,2-dichlorobenzene (DCB) at a concentration of 5 mg/mL, separately. The spray rate of P3HT:PC_61_BM solution was 0.075 mL·min^−1^ and the N_2_ carrier gas flow rate was held at a rate of 18 L·min^−1^. An ABL of S-WO_3_ was prepared by USP on P3HT:PC_61_BM blend film at 80 °C using N_2_ as carrier gas with a flow rate of 6 L·min^−1^, and the solution atomization rate was ~3 mL·min^−1^. The instrument used in USP is mentioned previously in Section 2.1. The ultrasonic transducer (JR-24, Siansonic) used aФ 20 mm piezoelectric vibrator at an ultrasonic frequency of 1700 KHz. AФ 20 mm nozzle was mounted 10 mm above the heating block. Precursor solutions were obtained by dissolving ammonium metatungstate ((NH_4_)_10_W_12_O_41_ 99.95%, Aladdin, Shanghai, China) powders into deionized water with a desired concentration. An E-WO_3_ ABL was deposited onto the substrate at a rate of 1–2 Å/S at a pressure of 5 × 10^−4^ Pa by multifunctional high vacuum film forming equipment (OLED-V Shenyang Vacuum Technology Institute). Subsequently, Ag anodes were finally deposited at a rate of about 10 Å/S under a pressure of 5 × 10^−4^ Pa without breaking the vacuum.

### 3.2. Measurement Method

The composition and electron structure of the ABLs were characterized by X-ray photoelectron spectroscopy (XPS, Thermo ESCALAB, Shanghai, China). The UPS measurements were performed with a He I (21.2 eV) discharge lamp (AXIS–NOVA System, Kratos, Manchester, UK). All samples were kept inside a high-vacuum chamber. TGA, DSC were taken using simultaneous thermal analyzer (STA 449 F3, Netzsch, Shanghai, China) with crucible (DSC/TG pan Al_2_O_3_), and the flow rate is 60 mL/min in nitrogen condition. The simple mass is ~5.7 mg, and the heating rate is 5 °C/min. The crystal structure was characterized by X-ray diffraction (XRD, X’Pert PRO, PANalytical, Cu K*α* radiation *λ* = 0.154056 nm, 40 kV and 40 mA) in grazing incidence mode. The Surface morphology of the ABLs, ITO and active layer was characterized by atomic force microscope (AFM, AFM 5500, Agilent, Tapping Mode, Chengdu, China). AFM images and RMS roughness are obtained by Gwyddion. Current density–voltage (*J*–*V*) curves were measured with Keithley 2400 under a xenon lamp solar simulator (7IS0503A, SOFN, Beijing, China) with an illumination power of 100 mW/cm^2^ [56]. Conductivity measurement and calculation procedure are shown in supplemental materials in Figure S4. A precision impedance analyzer (4294A, Agilent, Chengdu, China) was employed for impedance spectroscopy measurement. The range of measured frequency is from 40 Hz to 1 MHz, and 50 mV of modulation voltage without DC bias was used to extract the DC bias-dependent AC signal. All the measurements were carried out at ambient circumstance without encapsulation.

## 4. Conclusions

In summary, the low-temperature S-WO_3_ film shows low roughness, matched energy level, and high conductivity, which is suitable for the fabrication of OSCs with high charge transport efficiency responsible for the obviously increased *J_SC_* and *V_OC_*, and a higher PCE accordingly. The study of OSC performance processed from different concentrations of AT precursor shows that, when the concentration of AT precursor is 100 mg/L, the OSC has the highest PCE of 3.63%. Then, based on the characterization of S-WO_3_ film and mechanism analysis of USP method, the almost all-sprayed large area OSC with a PCE of ~1% was realized, which meets the essential prerequisite of roll-to-roll manufacturing and is compatible with large areas of a variety of thin film optoelectronics.

## Figures and Tables

**Table 1:** Comparison of device characteristics of OSCs based on E-WO_3_ and S-WO_3_ films with different precursor concentrations.

Devices	*V_OC_* (V)	*J_SC_* (mA·cm^−2^)	FF (%)	PCE (%)	R_S_ (Ω cm^2^)
20 nm E-WO_3_	0.57 ± 0.01	9.68 ± 0.09	58.73 ± 1.02	3.27 ± 0.11	1.06 ± 0.15
S-WO_3_ with concentration of AT					
25 mg/L	0.55 ± 0.03	9.58 ± 0.21	38.74 ± 2.62	2.03 ± 0.22	15.65 ± 1.76
100 mg/L	0.63 ± 0.02	10.45 ± 0.17	55.48 ± 1.27	3.63 ± 0.13	1.18 ± 0.31
200 mg/L	0.61 ± 0.02	10.12 ± 0.19	52.85 ± 1.36	3.27 ± 0.16	2.42 ± 0.73
300 mg/L	0.61 ± 0.02	9.52 ± 0.14	49.36 ± 0.95	2.86 ± 0.12	7.84 ± 0.58

**Table 2:** Parameters employed for the fitting of the impedance spectra by using an equivalent circuit model.

Devices	R1 (Ω)	C1 (F)	R2 (Ω)	C2 (F)	R3 (Ω)
20 nm E-WO_3_	3.32 × 10^5^	1.37 × 10^−9^	1547.00	9.60 × 10^−10^	220.20
S-WO_3_ with concentration of AT					
25 mg/L	1.78 × 10^6^	1.25 × 10^−9^	2049.00	6.983 × 10^−10^	245.90
100 mg/L	2.09 × 10^5^	2.50 × 10^−9^	850.80	1.16 × 10^−9^	107.50
200 mg/L	2.33 × 10^5^	3.49 × 10^−9^	1024.00	1.01 × 10^−9^	73.81
300 mg/L	4.08 × 10^5^	1.55 × 10^−9^	1682.00	8.76 × 10^−10^	160.30

## Supplementary Materials

The following are available online at www.mdpi.com/1996-1944/10/7/820/s1: Figure S1: J–V characteristics of OSCs with WO_3_ films deposited by vacuum thermal evaporation and spray-coated with different precursor concentration in air, Table S1: RMS of different films using in experiment.

## References

[1] Guenes S., Neugebauer H., Sariciftci N.S. (2007). Conjugated polymer-based organic solar cells. Chem. Rev..

[2] Yu J., Zheng Y., Huang J. (2014). Towards high performance organic photovoltaic cells: A review of recent development in organic photovoltaics. Polymers.

[3] Dennler G., Sariciftci N.S. (2005). Flexible conjugated polymer-based plastic solar cells: From basics to applications. Proc. IEEE.

[4] Huang J., Wang H., Yan K., Zhang X., Chen H., Li C., Yu J. (2017). Highly efficient organic solar cells consisting of double bulk heterojunction layers. Adv. Mater..

[5] Scharber M.C., Koppe M., Gao J., Cordella F., Loi M.A., Denk P., Morana M., Egelhaaf H.J., Forberich K., Dennler G. (2010). Influence of the bridging atom on the performance of a low-bandgap bulk heterojunction solar cell. Adv. Mater..

[6] Kan B., Zhang Q., Li M., Wan X., Ni W., Long G., Wang Y., Yang X., Feng H., Chen Y. (2014). Solution-processed organic solar cells based on dialkylthiol-substituted benzodithiophene unit with efficiency near 10%. J. Am. Chem. Soc..

[7] Yu G., Gao J., Hummelen J.C., Wudl F., Heeger A.J. (1995). Polymer photovoltaic cells: Enhanced efficiencies via a network of internal donor-acceptor heterojunctions. Science.

[8] Liu Y., Zhao J., Li Z., Cheng M., Ma W., Hu H., Jiang K., Lin H., Ade H., He Y. (2014). Aggregation and morphology control enables multiple cases of high-efficiency polymer solar cells. Nat. Commun..

[9] Zhao G., He Y., Li Y. (2010). 6.5% efficiency of polymer solar cells based on poly(3-hexylthiophene) and indene-C-60 bisadduct by device optimization. Adv. Mater..

[10] Zheng Y., Wu R., Shi W., Guan Z., Yu J. (2013). Effect of In Situ annealing on the performance of spray coated polymer solar cells. Sol. Energy Mater. Sol. Cells.

[11] Kong T., Wang H., Liu X., Yu J., Wang C. (2017). Improving the efficiency of bulk heterojunction polymer solar cells via binary-solvent treatment. IEEE J. Photovolt..

[12] Guo F., Li N., Fecher F.W., Gasparini N., Ramirez Quiroz C.O., Bronnbauer C., Hou Y., Radmiloviæ V.V., Radmiloviæ V.R., Spiecker E. (2015). A generic concept to overcome bandgap limitations for designing highly efficient multi-junction photovoltaic cells. Nat. Commun..

[13] Zhou Y., Fuenteshernandez C., Shim J.W., Khan T.M., Kippelen B. (2012). High performance polymeric charge recombination layer for organic tandem solar cells. Energy Environ. Sci..

[14] Li M., Gao K., Wan X., Zhang Q., Kan B., Xia R., Liu F., Yang X., Feng H., Ni W. (2016). Solution-processed organic tandem solar cells with power conversion efficiencies>12%. Nat. Photonics.

[15] Po R., Carbonera C., Bernardi A., Camaioni N. (2011). The role of buffer layers in polymer solar cells. Energy Environ. Sci..

[16] Li G., Chu C.W., Shrotriya V., Huang J., Yang Y. (2006). Efficient inverted polymer solar cells. Appl. Phys. Lett..

[17] Shrotriya V., Li G., Yao Y., Chu C.W. (2006). Transition metal oxides as the buffer layer for polymer photovoltaic cells. Appl. Phys. Lett..

[18] Sun Y., Takacs C.J., Cowan S.R., Seo J.H., Gong X., Roy A., Heeger A.J. (2011). Efficient, air-stable bulk heterojunction polymer solar cells using MoO_x_ as the anode interfacial layer. Adv. Mater..

[19] Chen C.P., Chen Y.D., Chuang S.C. (2011). High performance and highly durable inverted organic photovoltaics embedding solution processable vanadium oxides as an interfacial hole transporting layer. Adv. Mater..

[20] Shen L., Ruan S., Guo W., Meng F., Chen W. (2012). Semitransparent inverted polymer solar cells using MoO_3_/Ag/V_2_O_5_ as transparent anodes. Sol. Energy Mater. Sol. Cells.

[21] Shen L., Xu Y., Meng F., Li F., Ruan S., Chen W. (2011). Semitransparent polymer solar cells using V_2_O_5_/Ag/V_2_O_5_ as transparent anodes. Organ. Electron..

[22] Koo J.R., Lee S.J., Lee H.W., Lee D.H., Yang H.J., Kim W.Y., Kim Y.K. (2013). Flexible bottom-emitting white organic light-emitting diodes with semitransparent Ni/Ag/Ni anode. Opt. Express.

[23] Steirer K.X., Ndione P.F., Widjonarko N.E., Lloyd M.T., Meyer J., Ratcliff E.L., Kahn A., Armstrong N.R., Curtis C.J., Ginley D.S. (2011). Enhanced efficiency in plastic solar cells via energy matched solution processed NiO_x_ interlayers. Adv. Energy Mater..

[24] Go G.H., Shinde P.S., Doh C.H., Lee W.J. (2016). PVP-assisted synthesis of nanostructured transparent WO_3_ thin films for photoelectrochemical water splitting. Mater. Des..

[25] Gakhar R., Chidambaram D. (2016). Photoelectrochemical performance of zncdse sensitized WO_3_ thin films. Sol. Energy Mater. Sol. Cells.

[26] Li N., Stubhan T., Luechinger N.A., Halim S.C., Matt G.J., Ameri T., Brabec C.J. (2012). Inverted structure organic photovoltaic devices employing a low temperature solution processed WO_3_ anode buffer layer. Organ Electron..

[27] Choi H., Kim B., Ko M.J., Lee D., Kim H., Kim S.H., Kim K. (2012). Solution processed WO_3_ layer for the replacement of PEDOT:PSS layer in organic photovoltaic cells. Organ. Electron..

[28] Han S., Shin W.S., Seo M., Gupta D., Moon S., Yoo S. (2009). Improving performance of organic solar cells using amorphous tungsten oxides as an interfacial buffer layer on transparent anodes. Organ Electron..

[29] Zheng D., Huang J., Zheng Y., Yu J. (2015). High performance airbrush spray coated organic solar cells via tuning the surface tension and saturated vapor pressure of different ternary solvent systems. Organ. Electron..

[30] Zheng D., Huang W., Fan P., Zheng Y., Huang J., Yu J. (2017). Preparation of reduced grapheneoxide:ZnOhybrid cathode interlayer using In Situ thermal reduction/annealing for interconnecting nanostructure and its effect on organic solar cell. ACS Appl. Mater. Interfaces.

[31] Li H., Li S., Wang Y., Sarvari H., Zhang P., Wang M., Chen Z. (2016). A modified sequential deposition method for fabrication of perovskite solar cells. Sol. Energy.

[32] Hau S.K., Yip H., Jen A.K.Y. (2010). A review on the development of the inverted polymer solar cell architecture. Polym. Rev..

[33] Zhu X., Kawaharamura T., Stieg A.Z., Biswas C., Li L., Ma Z., Zurbuchen M.A., Pei Q., Wang K.L. (2015). Atmospheric and aqueous deposition of polycrystalline metal oxides using Mist-CVD for highly efficient inverted polymer solar cells. Nano Lett..

[34] Cheng J., Wang Q., Zhang C., Yang X., Hu R., Huang J., Yu J., Li L. (2016). Low-temperature preparationof ZnO thin film by atmospheric mist chemistry vapor deposition for flexible organic solar cells. J. Mater. Sci..

[35] Ji R., Cheng J., Yang X., Yu J., Li L. (2017). Enhanced charge carrier transport in spray-cast organic solar cells using solution processed MoO_3_ micro arrays. RSC Adv..

[36] Zhang J., Shi C., Chen J., Ying C., Wu N., Wang M. (2016). Pyrolysis preparation of WO_3_ thin films using ammonium metatungstate DMF/water solution for efficient compact layers in planar perovskite solar cells. J. Semicond..

[37] Sivakumar R., Moses Ezhil Raj A., Subramanian B., Jayachandran M., Trivedi D.C., Sanjeeviraja C. (2004). Preparation and characterization of spray deposited n-type WO_3_ thin films for electrochromic devices. Mater. Res. Bull..

[38] Chambon S., Derue L., Lahaye M., Pavageau B., Hirsch L., Wantz G. (2012). MoO_3_ Thickness, thermal annealing and solvent annealing effects on inverted and direct polymer photovoltaic solar cells. Materials.

[39] You L., Liu B., Liu T., Fan B., Cai Y., Guo L., Sun Y. (2017). Organic solar cells based on WO_2.72_ nanowire anode buffer layer with enhanced power conversion efficiency and ambient stability. ACS Appl. Mater. Interfaces.

[40] Wang G., Yang Y., Han D., Li Y. (2017). Oxygen defective metal oxides for energy conversion and storage. Nano Today.

[41] Guillain F., Tsikritzis D., Skoulatakis G., Kennou S., Wantz G., Vignau L. (2014). Annealing-free solution-processed tungsten oxide for inverted organic solar cells. Sol. Energy Mater. Sol. Cells.

[42] Lachkar A., Selmani A., Sacher E., Leclerc M., Mokhliss R. (1994). Metallization of polythiophenes I. interaction of vapor-deposited Cu, Ag and Au with poly(3-hexylthiophene) (P3HT). Synth. Met..

[43] Xu Z., Chen L.M., Yang G., Huang C.H., Hou J., Wu Y., Li G., Hsu C.S., Yang Y. (2009). Vertical phase separation in poly(3-hexylthiophene): Fullerene derivative blends and its advantage for inverted structure solar cells. Adv. Funct. Mater..

[44] Xu Z., Chen L.M., Chen M.H., Li G., Yang Y. (2009). Energy level alignment of poly(3-Hexylthiophene): [6,6]-Phenyl C61 butyric acid methyl ester bulk heterojunction. Appl. Phys. Lett..

[45] Meyer J., Hamwi S., Krger M., Kowalsky W., Riedl T., Kahn A. (2012). Transition metal oxides for organic electronics: Energetics, device physics and applications. Adv. Mater..

[46] Choi H., Ko S.J., Choi Y., Joo P., Kim T., Bo R.L., Jung J.W., Choi H.J., Cha M., Jeong J.R. (2013). Versatile surface plasmon resonance of carbon-dot-supported silver nanoparticles in polymer optoelectronic devices. Nat. Photonics.

[47] Mihailetchi V.D., Blom P.W.M., Hummelen J.C., Rispens M.T. (2003). Cathode dependence of the open-circuit voltage of polymer: Fullerene bulk heterojunction solar cells. J. Appl. Phys..

[48] Zhou Y., Fuentes-Hernandez C., Shim J., Meyer J., Giordano A.J., Li H., Winget P., Papadopoulos T., Cheun H., Kim J. (2012). A universal method to produce low-work function electrodes for organic electronics. Science.

[49] Murase S., Yang Y. (2012). Solution processed MoO_3_ interfacial layer for organic photovoltaics prepared by a facile synthesis method. Adv. Mater..

[50] Elumalai N.K., Uddin A. (2016). Open circuit voltage of organic solar cells: An in-depth review. Energy Environ. Sci..

[51] Yao E.P., Chen C.C., Gao J., Liu Y., Chen Q., Cai M., Hsu W.C., Hong Z., Li G., Yang Y. (2014). The study of solvent additive effects in efficient polymer photovoltaicsvia impedance spectroscopy. Sol. Energy Mater. Sol. Cells.

[52] Lang R.J. (1962). Ultrasonic atomization of liquids. J. Acoust. Soc. Am..

[53] Liu L., Wang M., Liu Y., Bi Q. (2015). Heat and mass transfer characteristics of evaporation and salt crystallization process of a saline droplet during depressurization. J. Ind. Eng. Chem..

[54] Viguie J.C., Spitz J. (1975). Chemical vapor deposition at low temperature. J. Electrochem. Soc..

[55] Li S., Zhang P., Chen H., Wang Y., Liu D., Wu J., Sarvari H., Chen Z.D. (2017). Mesoporous PbI_2_ assisted growth of large perovskite grains for efficient perovskite solar cells based on ZnO nanorods. J. Power Sources.

[56] Wang M., Li S., Zhang P., Wang Y., Li H., Chen Z. (2015). A modified sequential method used to prepare high quality perovskiteon ZnO nanorods. Chem. Phys. Lett..

