# Supplementary material for: Low-Temperature Preparation of Tungsten Oxide Anode Buffer Layer via Ultrasonic Spray Pyrolysis Method for Large-Area Organic Solar Cells
Source: Materials (Basel). 2017 Jul 18;10(7):820. doi: 10.3390/ma10070820 (PMC5551863; doi:10.3390/ma10070820)
Supplement: Supplementary file 1 [file materials-10-00820-s001.pdf]

SupplementaryMaterials: Low-temperature Preparation of tungsten trioxide Anode Buffer Layer with High Charge Transport Efficiency via the ultrasonic spray pyrolysis Method for Large-area Organic Solar Cells

Ran Ji [1,2], Ding Zheng [1], Chang Zhou [1], Jiang Cheng [2], Junsheng Yu[1,*] and Lu Li [2,*]

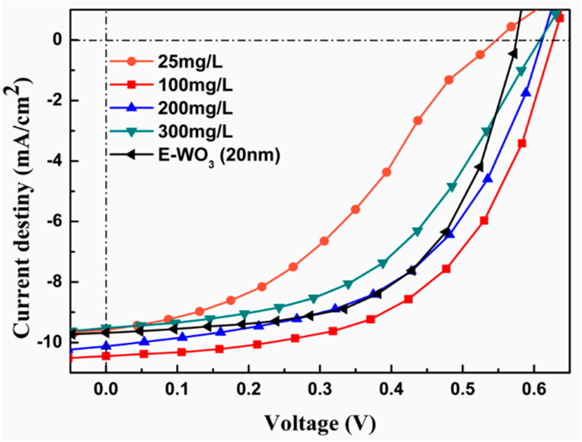

Figure S1. *J–V* characteristics of OSCs with E-WO_3 films and S-$WO_3$with different precursor concentrationin air.

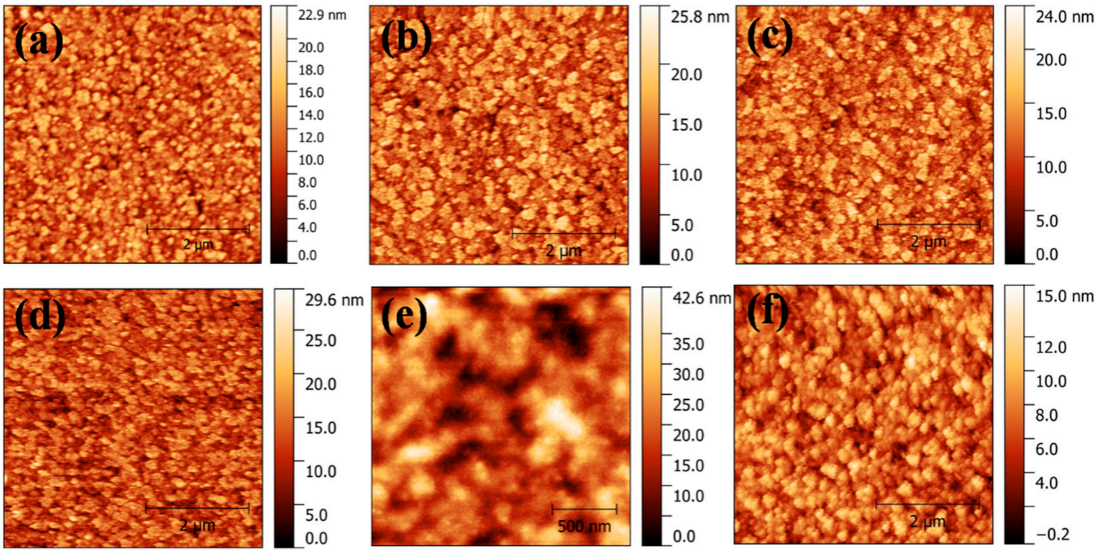

Figure S2. AFM images of S-WO_3 films with different AT concentration (**a**)25mg/L; (**b**)100mg/L; (**c**) 200mg/L; (**d**) 300 mg/L, and (**e**) active layers; (**f**) bare ITO.

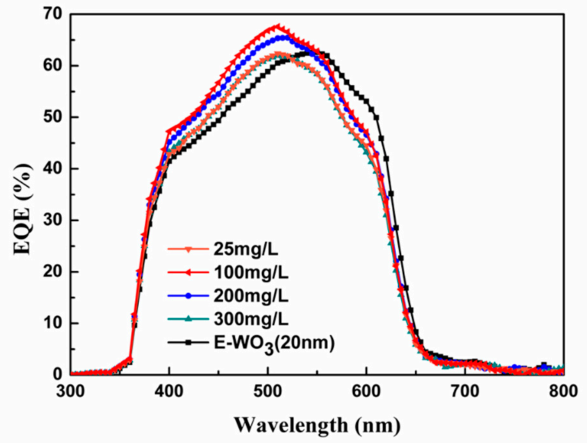

Figure S3.*EQE* characteristics of OSCs based on E-WO_3 film and S-WO_3 filmswith different precursor concentrationin air.

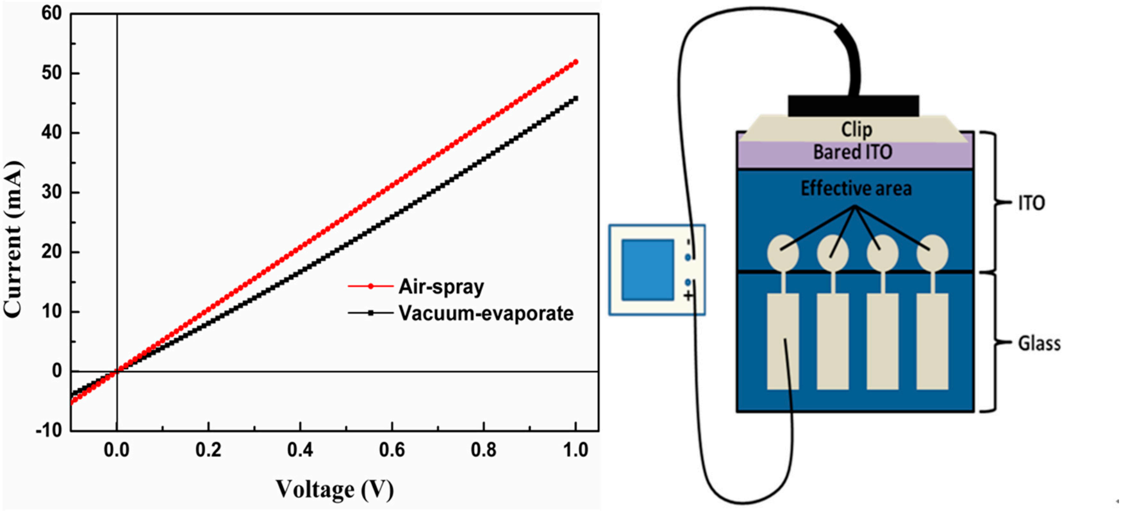

Figure S4.*I-V* curves of devices with ITO/ABL/Ag, and schematic diagram of conductivity test.

The conductivity of ABLs are calculated from the *I-V* data using the following Equation (1)

$$K = \frac{G \cdot L}{A} \tag{S1}$$

where K is the conductivity of ABL, and *G* is the conductance of the ABL, which is equal to the reciprocal of resistance. *L* is the thickness of ABL, and *A* is the cross-sectional area of ABL. The thickness of each ABLs is 20 nm, and the cross-sectional area of each ABLs is 0.03 cm^2. The conductivities of E-WO3 and S-WO3 are 3.05×10^{-4} S·m-1 and 5.17×10^{-4} $S{\cdot}m^{-1}$, respectively.

To get conductivity results with high repeatability, we compare the I-V curve of different devices, and these devices are in the same position of different glass sheet. The same position is apt to avoid the different resistance introduced by ITO. Besides, wire connected with silver electrode at a same position to prevent the different resistance introduced by silver. The result shows the same trend and miniscule difference in conductivity, and it proves that this method have a good repeatability.

TableS1. RMS of different films using in experiment.

ABL	25mg/L	100mg/L	200mg/L	300mg/L	E-WO_3	Bare ITO	Active layer
RMS (nm)	2.76	3.49	3.22	3.45	3.61	2.08	6.89
